# Ejaculation in testicular cancer patients after post-chemotherapy retroperitoneal lymph node dissection

**DOI:** 10.1038/sj.bjc.6690347

**Published:** 1999-04

**Authors:** K D Jacobsen, S Ous, H Wæhre, H Trasti, A E Stenwig, H H Lien, N Aass, S D Fosså

**Affiliations:** Departments of Medical Oncology and Radiotherapy, Surgical Oncology, Pathology, andRadiology, The Norwegian Radium Hospital, Oslo 0310, Norway

**Keywords:** testicular cancer, nerve-sparing RPLND, fertility

## Abstract

The purpose of this study was to evaluate fertility after different types of post-chemotherapy retroperitoneal lymph node dissection (RPLND). During 1980–1994, 192 patients with metastatic testicular cancer underwent post-chemotherapy RPLND with a gradual shift from modified bilateral template RPLND to nerve-sparing RPLND. Modified bilateral template RPLND was done in 92% of the patients operated during 1980–1984 as compared to 16% during 1989–1994. Pre- and post-treatment fertility was assessed by microscopic sperm analysis, determination of serum FSH and information on ejaculation and paternity. There was no significant difference of the survival rates between the three treatment periods. Antegrade ejaculation was preserved in 11% of the patients after modified bilateral template RPLND as compared to 89% after the nerve-sparing operation technique. The median ejaculatory volume decreased post-operatively, serum FSH increased and sperm density remained unchanged. Fifty-six patients attempted fatherhood after their treatment, and 27 fathered at least one child after an observation-time of 55 months, nine of them by assisted fertilization. Patients with initially advanced testicular cancer but limited residual retroperitoneal masses after induction chemotherapy can safely undergo limited post-chemotherapy RPLND as a part of multimodality treatment. After nerve-sparing RPLND antegrade ejaculation is preserved in 89% of the patients though the ejaculatory volume decreases after RPLND. Post-treatment fatherhood can be achieved in at least 50% of the patients attempting paternity. © 1999 Cancer Research Campaign


*British Journal of Cancer* (1999) **80**(1/2), 249–255
© 1999 Cancer Research Campaign
Article no. bjoc.1998.0347

### Ejaculation in testicular cancer patients after post-chemotherapy retroperitoneal lymph node dissection

**KD Jacobsen[1], S Ous[2], H Wæhre[2], H Trasti[2], AE Stenwig[3], HH Lien[4], N Aass[1] and SD Fosså[1]**

Departments of [1]Medical Oncology and Radiotherapy, [2]Surgical Oncology, [3]Pathology and [4]Radiology, The Norwegian Radium Hospital, 0310 Oslo, Norway

**Summary** The purpose of this study was to evaluate fertility after different types of post-chemotherapy retroperitoneal lymph node dissection (RPLND). During 1980–1994, 192 patients with metastatic testicular cancer underwent post-chemotherapy RPLND with a gradual shift from modified bilateral template RPLND to nerve-sparing RPLND. Modified bilateral template RPLND was done in 92% of the patients operated during 1980–1984 as compared to 16% during 1989–1994. Pre- and post-treatment fertility was assessed by microscopic sperm analysis, determination of serum FSH and information on ejaculation and paternity. There was no significant difference of the survival rates between the three treatment periods. Antegrade ejaculation was preserved in 11% of the patients after modified bilateral template RPLND as compared to 89% after the nerve-sparing operation technique. The median ejaculatory volume decreased post-operatively, serum FSH increased and sperm density remained unchanged. Fifty-six patients attempted fatherhood after their treatment, and 27 fathered at least one child after an observation-time of 55 months, nine of them by assisted fertilization. Patients with initially advanced testicular cancer but limited residual retroperitoneal masses after induction chemotherapy can safely undergo limited post-chemotherapy RPLND as a part of multimodality treatment. After nerve-sparing RPLND antegrade ejaculation is preserved in 89% of the patients though the ejaculatory volume decreases after RPLND. Post-treatment fatherhood can be achieved in at least 50% of the patients attempting paternity.

**Keywords:** testicular cancer; nerve-sparing RPLND; fertility

During recent years increasing attention has been devoted to long-term toxicity and quality of life of patients treated for testicular cancer (Moynihan, 1987; Roth et al, 1988; Aass et al, 1990; Bissett et al, 1990), fertility being an important dimension of psychosocial well-being and post-treatment morbidity.

Spermatogenesis is often transiently inhibited by modern standard chemotherapy (Drasga et al, 1983; Berthelsen, 1984; Brenner et al, 1985; Lange et al, 1987) with gradual recovery 10–18 months after discontinuation of chemotherapy. After retroperitoneal lymph node dissection (RPLND), ‘dry ejaculation’ represents a major risk of infertility, especially if this operation has to be done in patients with extended retroperitoneal metastases (Hermanek and Sigel, 1982; Wood et al, 1982; Lange et al, 1984; Fosså et al, 1991). Post-RPLND dry ejaculation is due to the resection of sympathetic nerves entering the superior hypogastric plexus (Jewett et al, 1988). After the original bilateral template RPLND almost all patients suffered from post-operative dry ejaculation, even if done as modified RPLND thus sparing the contralateral iliac region below the iliac bifurcation (Whitmore, 1982). Early in the 1980s unilateral template RPLND was developed (Fosså et al, 1984*a*; Pizzocaro et al, 1985; Weissbach et al, 1985). After this operation antegrade ejaculation remained in 80% of the patients. During the mid 1980s Donohue et al (1990) introduced nerve-sparing RPLND in the treatment of testicular cancer patients.

Unilateral and nerve-sparing RPLND techniques were primarily developed for patients with no or limited retroperitoneal tumour manifestations and were mostly applied as diagnostic means in testicular cancer patients without preceding chemotherapy. During the 1980s it became evident that more limited techniques of RPLND, such as unilateral (Fosså et al, 1985*a*) or nerve-sparing (Jewett et al, 1988) RPLND, might be sufficient. Some institutions even used unilateral RPLND techniques as post-chemotherapy surgical procedures. Other institutions performed resection of residual macroscopic tumours rather than a formal RPLND (Hendry et al, 1993).

In the present paper we summarize the Norwegian Radium Hospital’s (NRH) experience with post-chemotherapy RPLND from 1980 to 1994. The aim was to investigate the impact of different types of RPLND on ejaculatory function and thus on fertility.

### MATERIALS AND METHODS

### Patients

From 1980 to 1994 about 400 patients with testicular cancer underwent RPLND at the NRH, Oslo. A total of 192 operations were performed after cisplatin-based induction chemotherapy in patients presenting with metastases. Survival data are given for these 192 patients. The analysis of ejaculatory function and fertility comprises 174 men, excluding 13 patients who had died at the time of investigation (March 1996) and for whom the medical records did not contain any information about post-RPLND ejaculation (Table 1). Also excluded were four patients with severe psychological disturbances from whom no valid information could be obtained as regards ejaculatory function and one patient who was lost to follow-up without any information on ejaculatory function and fertility in his medical record. The patients’ initial staging

*Received 28 May 1998*
*Revised 24 October 1998*
*Accepted 13 October 1998*

*Correspondence to:* SD Fosså

**Table 1** Demographics

| | | |
|---|---|---|
| No. of patients | | |
| Total | | 174 |
| 1980–1984 | 33 | |
| 1985–1988 | 61 | |
| 1989–1994 | 80 | |
| Age (years) | | 27[1] (15–57)[2] |
| Side of the tumour | | |
| Right | | 78 |
| Left | | 96 |
| Prior testicular maldescent | | |
| Yes | | 17 |
| No | | 140 |
| Unknown | | 17 |
| Histology | | |
| TD | | 13 |
| MTI | | 66 |
| MTU | | 75 |
| MTT | | 15 |
| Seminoma | | 5 |
| Stage (at start of induction chemotherapy) | | |
| II | | 92 |
| III | | 18 |
| IV | | 64 |
| Maximum diameter of retroperitoneal mass (mm) | | |
| Before chemotherapy | | 46 (10–150) |
| After chemotherapy | | 22 (0–120) |
| No. of cycles induction chemoth. | | |
| 1–2 | | 2 |
| 3 | | 6 |
| 4 | | 134 |
| 5–6 | | 25 |
| > 6 | | 7 |
| Observation period (months) | | 52[1] (10–198)[2] |
| Relapse (overall) | | 17 |
| Retroperitoneal relapse | | 4 |

[1]Median; [2](range). TD = teratoma differentiated (teratoma); MTI = malignant teratoma intermediate (teratocarcinoma); MTU = malignant teratoma undifferentiated (embryonal carcinoma); MTT = malignant teratoma trophoblastic (choriocarcinoma).

was done according to the Royal Marsden classification system based on clinical examination, thoraco-abdominal computerized tomography (CT) and measurements of serum choriogonadotropin (HCG) and alpha fetoprotein (AFP).

### Treatment principles

Cisplatin-based induction chemotherapy was given to all patients with ≥ IIB non-seminomatous testicular cancer or ≥ IIC seminoma. Post-chemotherapy RPLND was to be done 4–10 weeks after induction chemotherapy in non-progressing patients who had initially presented with retroperitoneal tumour masses, even though these had completely regressed after cytostatic treatment as evidenced by abdominal CT (Fosså et al, 198*a*). Until about 1985 patients with advanced seminoma were included in post-chemotherapy RPLND treatment, but this policy was gradually abandoned thereafter (Fosså et al, 1987, 1988).

**Table 2** Year of retroperitoneal surgery and type of RPLND (A), stage (B) and size of retroperitoneal mass (C) (192 patients)

| | Year of surgery | | | |
|---|---|---|---|---|
| | **1980–1984** **63**[1] | **1985–1988** **42** | **1989–1994** **87** | **Total** **192** |
| A.Type of RPLND | | | | |
| Bilateral template | 58[1] (92%) | 27 (64%) | 14 (16%) | 99 |
| Unilateral template | 3 | 11 | 17 | 31 |
| Nerve-sparing | 2 | 4 | 56 | 62 |
| B.Tumour stage | | | | |
| II | 22 (35%) | 24 (57%) | 54 (62%) | 100 |
| III | 5 | 5 | 8 | 18 |
| IV | 36 | 13 | 25 | 74 |
| C. Largest diameter (mm) | | | | |
| Pre-chemotherapy | 31[2] (15–150)[3] | 35 (15–110) | 30 (10–136) | 46 (10–150) |
| Post-chemotherapy | 13 (0–120) | 14 (0–87) | 16 (0–110) | 22 (0–120) |

[1]Number of patients; [2]median; [3](range).

### Chemotherapy

From 1978 and the early 1980s, induction chemotherapy consisted of 4–6 cycles with the PVB regimen (cisplatin, vinblastine, bleomycin) (Narayan et al, 1982; Fosså et al, 1988; Aass et al, 1991). A few patients from 1980 to 1981 also received adriamycin as a component of consolidation treatment. From 1985, vinblastine was gradually substituted by VP-16 (bleomycin, etoposide, cisplatin [BEP]). From 1988, patients were included in international randomized trials evaluating the effect of carboplatin and ifosfamide in patients with germ cell tumours (Loehrer, 1990; Bajorin et al, 1993; Bosl and Motzer, 1997).

### Surgical procedures for RPLND

Three types of RPLND were carried out during the 15-year period (Table 2).

### *Modified bilateral template RPLND*

This operation was routine from 1980 to 1984 and followed the recommendations of Whitmore (1979): excluding the great vessels, all retroperitoneal soft tissue was excised as an anatomical template.

### *Unilateral template RPLND*

From 1985 to 1988, resection of retroperitoneal tissue was confined to the ipsilateral side of the aortic midline in patients with retroperitoneal tumour disease confined to one template.

During nerve-sparing RPLND the sympathetic nerve fibres were carefully dissected out, identified and preserved, at least on one side (Donohue et al, 1990). Unilateral template nerve-sparing RPLND (Figure 1) was introduced in 1992 by one of the authors (SO) for patients with tumours confined to the ipsilateral template.

Via a midline abdominal incision the peritoneum was incised from the foramen of Winslow, distally around the caecum and proximally, along the left side of the mesenteric root, to the superior mesenteric artery. The right colon and the mesentery of the small intestine were sharply mobilized off the thin fascia covering the retroperitoneal compartment and placed on the patient's ches

*© Cancer Research Campaign 1999*

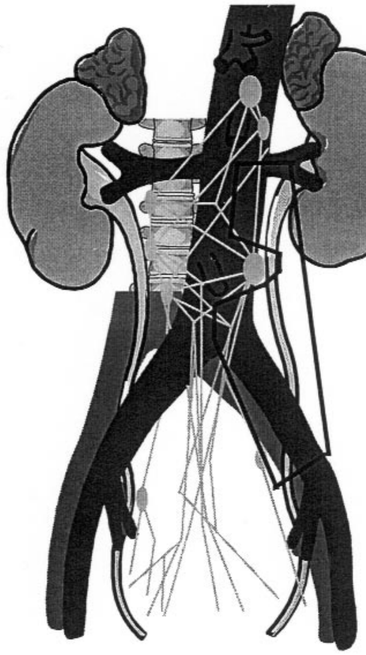

**Figure 1** Schematic presentation of the unilateral nerve-sparing RPLND. The pre-aortic sympathetic ganglion close to the origin of the inferior mesenteric artery is attempted to be preserved together with the hypogastric nerve bundle

The pancreas and the third part of duodenum were mobilized. With the left-sided template, the inferior mesenteric artery was routinely divided near to the aorta and the mesentery of the sigmoid colon was mobilized. The inferior mesenteric vein was usually preserved.

The nerve-sparing dissection commenced with the visual identification of the hypogastric nerves on the anterior surface of the aortic bifurcation, beneath the thin subperitoneal fascia covering aorta and the retroperitoneal compartment. The nerve bundles were safeguarded with a rubberband and followed proximally, until they diverged to their sides of origin. The landmark was usually identifiable at a ganglion at the pre-aortic level just distal to the origin of the inferior mesenteric artery. This ganglion was preserved. The nerve trunks belonging to the template of the ipsilateral template were removed. No further attempt was performed to dissect tissues on the opposite side.

Starting at the common iliac artery the tissue covering the great vessels was ligated, divided, mobilized and rolled off the circumference of the vessels proceeding stepwise proximally along the anterior midline of the vessels. Lumbal vessels were visualized, secured and divided. Following complete mobilization of the great vessels from the posterior abdominal wall within the appropriate template, all soft tissue was removed from the retroperitoneal space and the anterior spinous ligament. The root of the mesentery was repositioned and the peritoneum covering the posterior abdominal wall repaired with interrupted sutures.

### *Post-RPLND management*

No further treatment was given in patients with complete post-chemotherapy resections if the histology revealed complete necrosis/fibrosis or mature teratoma. Patients with viable cancer tissue in the resection specimen received three cycles of post-operative adjuvant chemotherapy (Hollender et al, 1997).

### *Follow-up*

All patients were seen at the NRH's outpatient department or at a local hospital with 2–6 months' intervals during the first 3–5 years, with no subsequent follow-up visits at the NRH. In all patients, a post-operative abdominal CT was performed about 2 months after RPLND. Routine follow-up investigations comprised clinical examination, measurements of serum HCG, AFP and regular chest X-rays. CT examinations were only performed when clinically indicated.

### Assessment of spermatogenesis and fertility

Before 1985 only those patients were assessed for pre-treatment fertility who spontaneously expressed plans for post-treatment paternity and in whom pre-treatment semen cryopreservation was considered. From 1985, information of testicular maldescent, previous fatherhood and involuntary infertility was routinely obtained. Evaluation of fertility was routinely done in all patients at the time of the initial referral to the NRH (2–3 weeks after unilateral orchiectomy, which usually was performed at the patient's local hospital). The serum hormone levels of luteinizing hormone (LH), follicle-stimulating hormone (FSH) and testosterone were measured. Light microscopic sperm cell analysis in the post-masturbation seminal fluid obtained after 3 days' sexual abstinence was done assessing sperm cell density ($\times 10^6$ $ml^{-1}$), mobility (grade 1–4) and motility (% mobile sperm cells). For the sake of simplification only sperm cell density will be considered in the present study. Whenever possible, serum hormone analyses and sperm analysis were repeated 1 and 3 years after discontinuation of all treatment. In addition, at the follow-up visits patients were to be asked about their post-operative ejaculatory function, their partners' eventual pregnancies and about involuntary infertility. If indicated, couples with infertility problems were referred to the University Infertility Clinic of the National Hospital, Oslo.

For the purpose of the study, a questionnaire was mailed to 165 living patients in March 1996 asking them for more recent information about post-treatment fatherhood, infertility and ejaculatory function. The patients were asked to categorize their ejaculation as 'normal' (antegrade), 'varying' (sometimes antegrade, sometimes 'dry ejaculation'), or as 'dry ejaculation' (never seminal fluid per urethram). The completed form, which represented the principal source of this study's outcome parameters, was returned by 155 patients (94%). For the ten patients who did not respond to the mailed questionnaire, and for nine patients who had died by the time of investigation, comparable information from the medical record was considered in the analysis.

### Statistical analysis

The data were analysed by the PC-version 6.0 of the SPSS using standard statistics (median, mean, range, $\chi^2$ test, Student's *t*-test). Due to the small number of paired results from sperm counts we decided to analyse all available pre- and post-treatment samples as independent parameters. Cancer-specific survival was assessed by the Kaplan–Meier method, evaluating differences between curves by the log-rank test. A $P$-value < 0.05 was regarded as statistically significant.

### RESULTS

### Performance of RPLND

During 1980–1984, 58 of 63 RPLNDs (92%) were performed as bilateral template operations (Table 2). This percentage had

© Cancer Research Campaign 1999

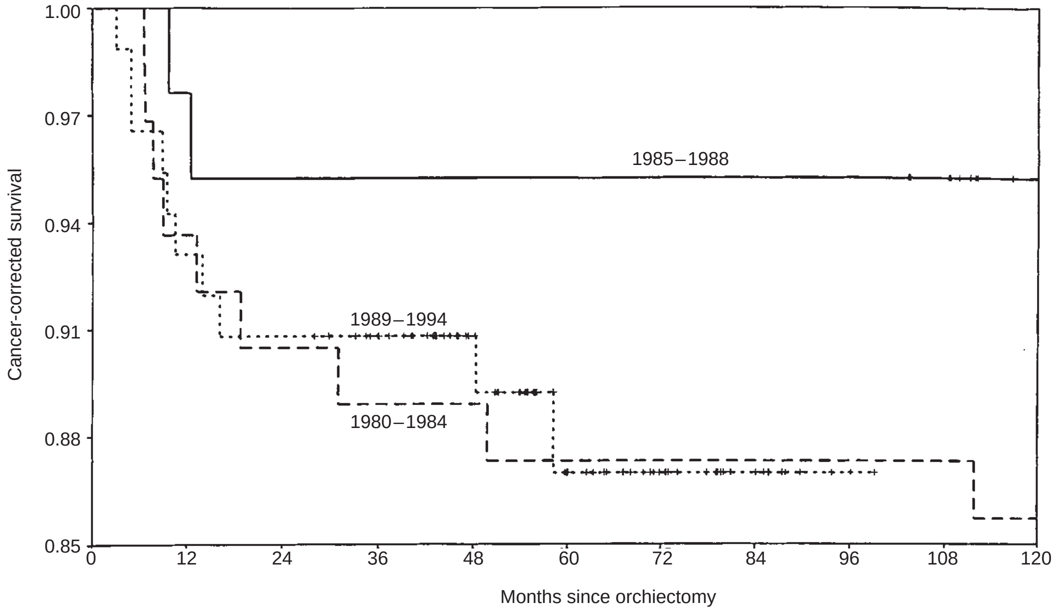

**Figure 2** Cancer-specific survival in patients with metastatic testicular cancer undergoing post-chemotherapy RPLND at the NRH (- – – –) 1980–1984, (——) 1985–1988, (. . . .) 1989–1994

decreased to 16% (14 of 87) during the third period. This reduction was not related to less advanced retroperitoneal tumour masses in the third period, though the percentage of stage II patients had increased gradually (Table 2B and C).

### Relapse and survival

In 30 of the 192 patients, the malignancy relapsed after RPLND. In 26 of these patients, residual viable cancer was found in the operation specimen; residual mature teratoma was demonstrated in four patients. In seven of these 26, recurrent retroperitoneal tumour masses were found at the time of relapse. Three of the seven patients had undergone modified bilateral template RPLND, one had had unilateral template RPLND and three patients were operated on by a nerve-sparing technique. The recurrent retroperitoneal tumours were resected in four patients, the histology showing viable malignant tumour tissue in one of them. In the three remaining patients with retroperitoneal relapse, all of them after nerve-sparing RPLND, the histology of the recurrent tumour was 'mature teratoma'. The former patient subsequently died of the germ cell malignancy, whereas the three other patients remain tumour-free. With a median observation time of 96 months (range 27–205) for surviving patients the cancer-specific survival was not significantly correlated with the period of RPLND (Figure 2).

Antegrade ejaculation was at least partially preserved in 17 of the 61 (28%) patients operated on during the first 5 years as compared to two of 33 patients (8%) and 63 of 80 patients (79%) in the second and third period respectively (Table 3).

Sixty-seven of 89 patients (75%) complained of dry ejaculation after bilateral modified template RPLND (Table 4). The comparable figures after nerve-sparing operations were three of 56 (5%). Dry ejaculation occurred in six of 29 patients (21%) operated on by unilateral template RPLND, three of them operated due to right-sided and three operated for left-sided tumours.

The median ejaculatory volume decreased from 4.4 ml before RPLND (64 patients) to 2.5 ml after the operation (26 patients) ($P < 0.001$, Table 5). With the reservation of small numbers, the reduction of the ejaculatory volume was least after nerve-sparing RPLND as compared to the template RPLND techniques without nerve-sparing. The sperm cell density remained unchanged.

Fifty-six of the 157 evaluable patients (36%) attempted to achieve fatherhood after their treatment (Table 6). Of the 35 patients with antegrade/variable ejaculation 18 were successful, two of them by fertilization using the patient's post-treatment sperm cells. Only nine of the 21 patients with dry ejaculation who planned paternity achieved fatherhood after their treatment, seven patients by assisted fertilization using the pre-treatment sperm cells from the sperm bank, and two patients after treatment with sympathomimetic agents.

**Table 3** Ejaculation and years for RPLND

| | Year of surgery | | | |
|---|---|---|---|---|
| Ejaculation | 1980–1984 | 1985–1988 | 1989–1994 | Total |
| Antegrade | 17 (28%) | 2 (6%) | 63 (79%) | 82 |
| Variable | 5 (8%) | 5 (15%) | 6 (7%) | 16 |
| 'Dry' | 39 (64%) | 26 (79%) | 11 (14%) | 76 |
| Total | 61 | 33 | 80 | 174 |

**Table 4** Ejaculation and type of RPLND

| | Type of surgery | | | |
|---|---|---|---|---|
| Ejaculation | Modified bilateral template | Unilateral template | Nerve-sparing | Total |
| Antegrade | 10 (11%) | 22 (76%) | 50 (89%) | 82 |
| Variable | 12 (13%) | 1 (3%) | 3 (5%) | 16 |
| 'Dry' | 67 (75%) | 6 (21%) | 3 (5%) | 76 |
| Total | 89 | 29 | 56 | 174 |

 © Cancer Research Campaign 1999

**Table 5** Sperm analysis

| RLND type | Pre-treatment | Post-treatment | *P*-value |
|---|---|---|---|
| Bilateral template | | | |
| | 24 patients | 5 patients | |
| Volume (ml) | 3.9[1] (1.0–9.7)[2] | 2.3 (1.7–4.0) | 0.04 |
| Density ($\times 10^6$ ml$^{-1}$) | 14 (0–75) | 12.5 (0–35) | NS |
| Unilateral template | | | |
| | 10 patients | 6 patients | |
| Volume (ml) | 5.7 (1.4–11.3) | 2.6 (0.7–5.3) | 0.03 |
| Density ($\times 10^6$ ml$^{-1}$) | 27.5 (2–61) | 17.5 (1–99) | NS |
| Nerve-sparing | | | |
| | 30 patients | 15 patients | |
| Volume (ml) | 3.3 (0.5–6.3) | 2.8 (0.9–6.6) | 0.03 |
| Density ($\times 10^6$ ml$^{-1}$) | 16.5 (0–143) | 38 (0–869) | NS |
| All | | | |
| | 64 patients | 26 patients | |
| Volume (ml) | 4.4 (0.5–11.3) | 2.5 (0.7–6.6) | < 0.001[3] |
| Density ($\times 10^6$ ml$^{-1}$) | 16.5 (0–76) | 25.0 (0–99) | NS |

[1]Median; [2]range; [3]Wilcoxon rank test.

**Table 6** Fertility and ejaculatory function

| | Antegrade/variable (98 patients) | 'Dry ejaculation' (76 patients) |
|---|---|---|
| Age at orchiectomy (years) | 27 ± 8[1] | 27 ± 9 |
| Prior testicular maldescent | 12 | 5 |
| Observation time (months) | 77 ± 41 | 133 ± 46 |
| No. of cycles | | |
| < 4 | 4 | 4 |
| 4 | 74 | 60 |
| > 4 | 20 | 12 |
| No. of children before orchiectomy | | |
| 0 | 60 | 38 |
| 1 | 11 | 12 |
| 2 | 12 | 13 |
| > 2 | 7 | 4 |
| Unknown | 8 | 9 |
| FSH after treatment (U l$^{-1}$) | 15.4 ± 11 | 17.7 ± 14 |
| Attempted fatherhood after treatment/evaluable | 35/90 | 21/67 |
| Achieved paternity (≥ × 1) | 18 (2)[2] | 9 (7)[3] (2)[4] |
| No. of paternities after treatment | | |
| 1 | 14 | 9[5] |
| 2 | 3 | |
| 6 | 1 | |
| No. of patients with attempts of assisted fertilization | 2 | 10 |

[1]Mean ± standard deviation; [2,3,4]Number of paternities achieved by assisted fertilization with fresh[2] or frozen[3] semen or after sympathomimetic agents[4]; [5]Twins in two cases and triplets in one patient.

### DISCUSSION

In a prospective study from Norway (Aass and Fosså, 1988) 67% of the patients, at the time of diagnosis of testicular cancer, stated that they definitely planned to have children after their treatment, especially the younger non-seminoma patients.

Several investigators have shown the time-dependent recovery of spermatogenesis in 60–80% of the patients treated with platin-based chemotherapy (Fosså et al, 1985*b*; Lampe et al, 1997). The preservation of patients' ejaculatory function thus becomes a major issue for maintenance of fertility. Furthermore, for many men, the experience of antegrade ejaculation represents an important component of an undisturbed sexual life. The present paper therefore predominantly deals with ejaculatory function 1 year or later after RPLND, assessed by the volume of the ejaculate. Secondarily, we also evaluated the sperm count, though these results are dependent more on the type of chemotherapy than of RPLND. Contrary to ejaculatory function, sperm cell density also improves with time after the first post-treatment year.

Post-chemotherapy RPLND continues to be an important approach in the management of testicular cancer in Norway even in patients with a normal abdominal CT. The necrotic/fibrotic tissue may include microscopic foci of mature teratoma (Fosså et al, 1984*b*, 1992). The general belief is that complete resection of this histological subtype is essential for long-time progression-free survival. Foci of vital malignant germ cell tumour may also be found (Fosså et al, 1992).

Initial RPLND techniques focused on complete removal of all lymphatic tissue. Loss of emission and antegrade ejaculation, and thus infertility, were the main late side-effects after post-chemotherapy bilateral template RPLND as performed in the early 1980s. With a better understanding of the pattern of the lymphatic spread of testicular cancer more limited RPLND techniques have been developed. The preservation of the hypogastric plexus and the inferior mesenteric ganglion is of particular importance for the maintenance of antegrade ejaculation and preservation of neural continuity of at least one side saves antegrade ejaculation.

Antegrade ejaculation is thus preserved in 80% of the patients after unilateral template RPLND more often after right-sided than left-sided RPLND (Lange et al, 1984; Fosså et al, 1984*a*, 1985*a*, 1988).

Nerve-sparing RPLND was initially introduced as an alternative technique for primary surgical staging of low-stage non-seminoma. Donohue et al (1990) attempted to identify the sympathetic fibres emerging from the thoraco-lumbal ganglia on both sides and to preserve their continuity by thorough dissection of the retroperitoneal lymphatic tissue surrounding the nerves. At least 90% of patients operated on with this technique preserved antegrade ejaculation. Subsequently, the above technique was also applied during post-chemotherapy RPLND in patients with limited residual retroperitoneal masses (Donohue et al, 1990). This accounts to about two-thirds of the patients requiring RPLND after induction chemotherapy.

The technique used at the NRH after 1992 represents a modification of Donohue et al's original technique and is attempted only if limited tumour masses are confined to one side. Emphasis is put on the preservation of the hypogastric plexus and the inferior mesenteric ganglion together with the preservation of neuro-continuity of the sympathetic fibres on the non-tumour-bearing side. This is achieved by omission of any resection of the contralateral side's lymphatic tissue. The lymphatic tissue on the tumour-bearing side is removed with a subsequent template resection proximally to the inferior mesenteric ganglion. In experienced hands the complete operation usually takes 2–3 h. Only in extra-ordinary cases, with very limited residual tumour masses on both sides, is resection of the lymphatic tissue on both sides performed. Identification and preservation of the bilateral sympathetic fibres is in these cases attempted, thus following the original recommendations for nerve-sparing technique (Donohue et al, 1990).

© Cancer Research Campaign 1999

The policy of performing nerve-sparing post-chemotherapy RPLND, whenever possible, has been followed by antegrade or variable ejaculation in 86% of the patients operated from 1989 to 1994, and antegrade ejaculation is preserved in 95% of the patients by nerve-sparing technique. However, the seminal fluid volume decreases as compared to the pre-treatment situation. The fact that with a median observation time of 55 months about one-third of the patients have attempted fatherhood underlines the clinical importance of preservation of antegrade ejaculation. An additional advantage of preserved antegrade ejaculation is that the patients' post-treatment semen may be used for assisted fertilization if fatherhood can not be achieved by 'natural means'.

Hendry et al (1992) had similar favourable results as concerns preservation of antegrade ejaculation when removing a residual mass rather than performing a formal RPLND. No data are so far available which would indicate the superiority of either treatment. At present we recommend institutions to choose the type of operation they feel most confident with.

After post-chemotherapy nerve-sparing RPLND there might be slightly increased risk of a retroperitoneal recurrence. Safe follow-up of these patients is essential and requires the close cooperation between clinicians and radiologists. The interpretation of post-RPLND abdominal CTs requires thorough knowledge of the typical visualization of the post-operative anatomy. The performance of unilateral template RPLND, but in particular of nerve-sparing RPLND, requires experience in the technical aspects of the operation, understanding of the tumour biology of advanced testicular cancer after induction chemotherapy and a close cooperation with the responsible oncologist and pathologist. Only major cancer centres will probably gain sufficient experience which lead to optimal results.

The present report leads to the following conclusions:

1. Modern treatment of chemotherapy of testicular cancer has to take into account that at least one-third of the patients want to father a child after their treatment.
2. Post-chemotherapy RPLND can safely be done by nerve-sparing techniques in most patients with limited residual retroperitoneal tumour masses.
3. Nerve-sparing post-chemotherapy RPLND preserves antegrade ejaculation in about 90% of the patients and enables post-treatment paternity in at least 50% of those who attempt fatherhood after their treatment.
4. The close cooperation between oncologists, surgeons, pathologists and radiologists is a condition sine qua non in the fertility-preserving approach nerve-sparing RPLND.

### ACKNOWLEDGEMENTS

The authors want to thank John P Donohue, MD, PhD for introducing the nerve-sparing operation technique for the surgical staff. The Norwegian Cancer Society is acknowledged for financing the project.

### REFERENCES

Aass N and Fosså SD (1988) Paternity in young patients with testicular cancer expectations and experience. *Prog Clin Biol Res* **269**: 481–491

Aass N, Fosså SD, Aas M and Lindegaard MW (1990) Renal function related to different treatment modalities for malignant germ cell tumours. *Br J Cancer* **62**: 842–846

Aass N, Fosså SD, Theodorsen L and Norman N (1991) Prediction of long-term gonadal toxicity after standard treatment for testicular cancer. *Eur J Cancer* **27**: 1087–1091

Bajorin DF, Sarosdy MF, Pfister DG, Mazumdar M, Motzer RJ, Scher HI, Geller NL, Fair WR, Herr H, Sogani P, Sheinfeld J, Russo P, Vlamis V, Carey R, Vogelzang NJ, Crawford ED and Bosl GJ (1993) Randomized trial of etoposide and cisplatin versus etoposide and carboplatin in patients with good-risk germ cell tumors: a multiinstitutional study. *J Clin Oncol* **11**: 598–606

Berthelsen JG (1984) Sperm counts and serum follicle-stimulating hormone levels before and after radiotherapy and chemotherapy in men with testicular germ cell cancer. *Fertil Steril* **41**: 281–286

Bissett D, Kunkeler L, Zwaneburg L, Paul J, Gray C, Swan IRC, Kerr DJ and Kaye SB (1990) Long-term sequelae of treatment for testicular germ cell tumours. *Br J Cancer* **62**: 655–659

Bosl GJ and Motzer RJ (1997) Testicular germ-cell cancer. Review Art. *N Engl J Med* **337**: 242–253

Brenner J, Vugrin D and Whitmore WF Jr (1985) Effect of treatment on fertility and sexual function in males with metastatic nonseminomatous germ cell tumors of testis. *Am J Clin Oncol* **8**: 178–182

Donohue JP, Foster RS, Rowland RG, Bihrle R, Jones R and Geier G (1990) Nervesparing retroperitoneal lymphadenectomy with preservation of ejaculation. *J Urol* **144**: 287–292

Drasga RE, Einhorn LH, Williams SD, Patel DN and Stevens EE (1983) Fertility after chemotherapy for testicular cancer. *J Clin Oncol* **1**: 179–183

Fosså SD, Klepp O, Ous S, Lien HH, Stenwig AE, Åbyholm T and Kaalhus O (1984*a*) Unilateral retroperitoneal lymph node dissection in patients with nonseminomatous testicular tumor in clinical stage. I. *Eur Urol* **10**: 17–23

Fosså SD, Klepp O, Ous S, Lien HH, Stenwig JT, Abeler V, Eliassen G and H∅st H (1984*b*) Multi-modality treatment in males with advanced malignant germ cell tumours. II. Experience with surgery and radiotherapy following cisplatinum-based chemotherapy. *Scand J Urol Nephrol* **18**: 21–26

Fosså SD, Ous S, Åbyholm T and Loeb M (1985*a*) Post-treatment fertility in patients with testicular cancer. I. Influence of retroperitoneal lymph node dissection on ejaculatory potency. *Br J Urol* **57**: 204–209

Fosså SD, Ous S, Åbyholm T, Norman N and Leob M (1985*b*) Post-treatment fertility in patients with testicular cancer. II. Influence of cisplatin-based combination chemotherapy and of retroperitoneal surgery on hormone and sperm cell production. *Br J Urol* **57**: 210–214

Fosså SD, Borge L, Aass N, Johannessen NB, Stenwig AE and Kaalhus O (1987) The treatment of advanced metastatic seminoma: Experience in 55 cases. *J Clin Oncol* **5**: 1071–1077

Fosså SD, Aass N and Kaalhus O (1988) Testicular cancer in young Norwegians. *Surg Oncol* **39**: 43–63

Fosså SD, Aass N, Ous S and Waehre H (1991) Long-term morbidity and quality of life in testicular cancer patients. *Scand J Urol Nephrol Suppl* **138**: 241–246

Fosså SD, Qvist H, Stenwig AE, Lien HH, Ous S, Giercksky KE (1992) Is postchemotherapy retroperitoneal surgery necessary in patients with non-seminomatous testicular cancer and minimal residual tumor masses? *J Clin Oncol* **10**: 569–573

Hendry WF, A'Hern RP, Hetherington JW, Peckham MJ, Dearnaley DP and Horwich A (1993) Para-aortic lymphadenectomy after chemotherapy for metastatic non-seminomatous germ cell tumours: Prognostic value and therapeutic benefit. *Br J Urol* **71**: 208–213

Hermanek P and Sigel A (1982) Necessary extent of lymph node dissection in testicular tumours. A histopathological investigation. *Eur Urol* **8**: 135–144

Hollender Å, Stenwig EA, Ous S and Fosså SD (1997) Survival of patients with viable malignant non-seminomatous germ cell tumour persistent after cisplatin-based induction chemotherapy. *Eur Urol* **31**: 141–147

Jewett MAS, Kong YSP, Goldberg SD, Sturgeon JFC, Thomas GM, Alison RE and Gospodarowicz MK (1988) Retroperitoneal lymphadenectomy for testis tumor with nerve sparing for ejaculation. *J Urol* **139**: 1220–1224

Lampe H, Horwich A, Norman A, Nicholls J and Dearnaley DP (1997) Fertility after chemotherapy for testicular germ cell cancers. *J Clin Oncol* **15**: 239–245

Lange PH, Narayan P and Fraley EE (1984) Fertility issues following therapy for testicular cancer. *Sem in Urol* **2**: 264–274

Lange PH, Chang WY and Fraley EE (1987) Fertility issues in the therapy of non-seminomatous testicular tumors. *Urol Clin North Am* **14**: 731–747

Loehrer PJ Sr (1990) Ifosfamide in testicular cancer. *Semin Oncol* **17**: 2–5

Moynihan C (1987) Testicular cancer: the psychosocial problems of patients and their relatives. *Cancer Surveys* **6**: 477–510

Narayan P, Lange PH and Fraley EE (1982) Ejaculation and fertility after extended retroperitoneal lymph node dissection for testicular cancer. *J Urol* **127**: 685–688

 © Cancer Research Campaign 1999

Pizzocaro G, Salvioni R and Zanoni F (1985) Unilateral lymphadenectomy in intraoperative stage I non-seminomatous germinal testis cancer. *J Urol* **134**: 485–489

Roth BJ, Greist A, Kubilis PS, Williams SD and Einhorn LH (1988) Cisplatin-based combination chemotherapy for disseminated germ cell tumors: long-term follow-up. *J Clin Oncol* **6**: 1239–1247

Weissbach L, Boedefeld EA and Oberdörster W (1985) Modified RLND as a means to preserve ejaculation. In: *Testicular Cancer*, Khoury S, Kuss R, Murphy GP, et al (eds), pp. 323–334. Alan R. Liss: New York

Whitmore WF Jr (1979) Surgical treatment of adult germinal testis tumors. *Semin Oncol* **6**: 55–68

Whitmore WF Jr (1982) Surgical treatment of clinical stage I nonseminomatous germ cell tumors of the testis. *Cancer Treat Rep* **66**: 5–10

Wood DP, Herr HW, Heller G, Vlamis V, Sogani PC, Motzer RJ, Fair WR and Bosl GJ (1982) Distribution of retroperitoneal metastases after chemotherapy in patients with non-seminomatous germ cell tumors. *J Urol* **148**: 1812–1816

*© Cancer Research Campaign 1999*
